# DNA Methylation Silences Exogenous Gene Expression in Transgenic Birch Progeny

**DOI:** 10.3389/fpls.2020.523748

**Published:** 2020-12-22

**Authors:** Minghao Ma, Xiaohui Chen, Yibo Yin, Ruixin Fan, Bo Li, Yaguang Zhan, Fansuo Zeng

**Affiliations:** ^1^Key Laboratory of Saline-Alkali Vegetation Ecology Restoration (Northeast Forestry University), Ministry of Education, Harbin, China; ^2^College of Life Science, Northeast Forestry University, Harbin, China

**Keywords:** transgenic *Betula platyphylla*, genetic stability, genetically modified silencing, DNA methylation, insect resistance

## Abstract

The genetic stability of exogenous genes in the progeny of transgenic trees is extremely important in forest breeding; however, it remains largely unclear. We selected transgenic birch (*Betula platyphylla*) and its hybrid F1 progeny to investigate the expression stability and silencing mechanism of exogenous genes. We found that the exogenous genes of transgenic birch could be transmitted to their offspring through sexual reproduction. The exogenous genes were segregated during genetic transmission. The hybrid progeny of transgenic birch WT1×TP22 (184) and WT1×TP23 (212) showed higher *Bgt* expression and greater insect resistance than their parents. However, the hybrid progeny of transgenic birch TP23×TP49 (196) showed much lower *Bgt* expression, which was only 13.5% of the expression in its parents. To elucidate the mechanism underlying the variation in gene expression between the parents and progeny, we analyzed the methylation rates of *Bgt* in its promoter and coding regions. The hybrid progeny with normally expressed exogenous genes showed much lower methylation rates (0–29%) than the hybrid progeny with silenced exogenous genes (32.35–45.95%). These results suggest that transgene silencing in the progeny is mainly due to DNA methylation at cytosine residues. We further demonstrated that methylation in the promoter region, rather than in the coding region, leads to gene silencing. We also investigated the relative expression levels of three methyltransferase genes: *BpCMT, BpDRM*, and *BpMET*. The transgenic birch line 196 with a silenced *Gus* gene showed, respectively, 2.54, 9.92, and 4.54 times higher expression levels of *BpCMT, BpDRM*, and *BpMET* than its parents. These trends are consistent with and corroborate the high methylation levels of exogenous genes in the transgenic birch line 196. Therefore, our study suggests that DNA methylation in the promoter region leads to silencing of exogenous genes in transgenic progeny of birch.

## Introduction

Exogenous genes can be randomly integrated into the recipient chromosome through homologous or nonhomologous recombination using plasmid vectors. The main problems in the improvement of genetic traits of tree species are the stability of exogenous genes in the host cells and the heritability of improved traits to the F1 generation (Creux et al., 2013). There are many studies on the genetic stability of transgenic plants (Mathieu et al., 2007; Prokopuk et al., 2015). Among these, however, only a few studies reveal the genetic stability of exogenous genes in woody plants with a long growth cycle. Ren et al. (2017) report successful the transfer of exogenous genes into progenies using transgenic poplars as parent plants for sexual hybridization to breed new insect-resistant varieties. Analysis of the genetic stability of *in vitro* propagated plants of “Jaspi” (a clonal rootstock of *Prunus*) shows almost no somaclonal variation among the micropropagated plants (Mahajan et al., 2017). A study on the development of secondary somatic embryos and genetic stability of regenerated *Hevea brasiliensis* plants indicates that the genome remained stable during multiplication (Wang et al., 2017). Cervera et al. (1998) conducted a follow-up survey of genetically modified citrus for 5 years and find that the *Gus* gene could be stably expressed without exogenous gene silencing. However, transgenes in genetically modified plants are often not stably expressed during development or in subsequent generations (Dietz-Pfeilstetter et al., 2016). Exogenous genes are frequently silenced in contemporary transformants or their progeny. The stability of integration and expression levels of transgenes in long-term micropropagation clones of transgenic birch (*Betula Platyphylla*) have also been examined. Transcriptional gene silencing (TGS) occurs in regenerated transgenic lines (Zeng et al., 2010a) although the silenced genes could be reactivated by treatment with 50–200 μM 5-azacytidine (Azac). Moreover, a decrease in expression level with an increasing number of subcultures is reportedly associated with DNA methylation (Zeng et al., 2010a).

Gene silencing is an important mechanism to regulate gene expression. It is a defense mechanism of organisms at the level of gene regulation. It commonly occurs during exogenous DNA invasion, viral infection, and DNA transposition and rearrangement (Arnaud et al., 2000; Seymour et al., 2014). There are many mechanisms underlying exogenous gene silencing, including that some siRNA-mediated T-DNA insertion mutants induce homology-dependent silencing of the 35S promoter (homology-dependent gene silencing, HDGS) (Mlotshwa et al., 2010). Potential factors affecting HDGS include the degree of similarity between the transgene and the endogenous gene, the complexity of the host genome, the location of the transgene, and the type of transgene promoter and terminator, etc. Condensation of chromatin or degradation of transcripts by a different mechanism can also cause inactivation/silencing of transgene activity (Fagard and Vaucheret, 2000). Chromosome position and flanking host DNA negatively regulate exogenous gene expression (Matzke et al., 2000). One of the main mechanisms is DNA methylation (Sijen et al., 2001; Miao et al., 2020). Methylation usually modifies the cytosine residues in the CpG structure to yield 5-methylcytosine (5-mC), which changes the local conformation of DNA, affects the interaction between protein and DNA, and disrupts transcriptional activity. As an important epigenetic modification, DNA methylation is prevalent in eukaryotic and prokaryotic organisms and efficiently regulates gene function (Kako et al., 2019; Pei et al., 2019). Peerbolte et al. (1986) first discovered that the gene on the T-DNA of *Agrobacterium tumefaciens* was methylated in transgenic tobacco, and its expression was inhibited. Low cytosine content in the vector sequence effectively reduced the methylation of exogenous genes and improved their expression efficiency (Grunau et al., 2001). High DNA methylation inhibits gene expression, ultimately resulting in gene silencing (Pierard et al., 2010; Lou et al., 2014). In transgenic cotton, Verkest et al. (2019) find that transgenic plants stably expressing exogenous genes show little or no methylation, whereas plants with silenced exogenous genes show hypermethylation in the promoter and coding regions.

DNA methylation modification is closely related to the activity of DNA methyltransferases. In plants, DNA methylation occurs in the symmetric CG and CHG sequence contexts as well as in the asymmetric CHH sequence contexts (H represents A, T, or C) (Huang et al., 2019). Various mechanisms are involved in establishing, maintaining, and removing the DNA methylation mark. CG, CHG, and CHH methylation are maintained through mechanisms involving DNA methyltransferases, including Methyltransferase 1 (MET1), Chromomethylase 3 (Huang et al., 2019), and domain-rearranged methyltransferases (DRM1 and DRM2) (Matzke and Mosher, 2014).

Overall, it is important to study the expression characteristics and methylation patterns of exogenous genes in transgenic perennial trees and their progeny (Nagle et al., 2018). Birch is one of the most important commercial tree species in Northeastern China, and it is the most prevalent hardwood. Several species of forest insect pests heavily attack birch (Zeng et al., 2009). Previously, we successfully obtained transgenic insect-resistant birch plants and their hybrid progeny. In this study, we aimed to (1) assess the genetic stability of exogenous genes in the progeny of transgenic birch; (2) reveal the effects of DNA methylation on exogenous gene expression using DNA methylation analysis of *Bgt* and *Gus*; and (3) explore the relationships among the expression of methyltransferases, level of DNA methylation, and expression of exogenous genes.

## Materials and Methods

### Transgenic Birch Pollen Collection and Hybridization

Transgenic birch plants were developed via *Agrobacterium* (LBA4404 strain) mediated transformation. The transformation vector was pCAMBIA-2301, which contained the selectable marker gene *Npt* II; the reporter gene *Gus*; and the fused *Bgt* gene, comprising an insecticidal toxin gene from a spider (*Atrax robustus*) and the C terminal of Cry IA (b) from *Bacillus thuringiensis*. Transgenic plants were grown by *in vitro* propagation and then, together with nontransformed control plants, cultivated in a greenhouse under natural daylight conditions (Zeng et al., 2010b).

Flowering branches of transgenic birch plants were collected at the end of April for controlled hydroponics. Pollen grains were collected, dried, sealed, and stored at 4°C. For intensive breeding, birch pollen grains were collected as the male parent. Birch plants with female flowers were emasculated and bagged as the female parent for hybridization before being pollinated. Pollination was conducted with a duster, twice a day, for four consecutive days. Hybrid combinations were as follows: WT×TP73, TP73×WT, TP73×TP22, WT×TP22, TP22×TP22, TP23×TP49, WT×TP23, and TP23×WT. Gene integration and expression characteristics of transgenic birch parents are summarized in Table 1 (Zeng et al., 2009, 2010b). The hybrid combinations and lines of the progeny are listed in Table 2.

**Table 1:** Copy number and expression of exogenous genes.

**Number**	***Gus***			***Bgt***			***Npt II***
	**Copies**	**Transcriptional expression**	**Protein**	**Copies**	**Transcriptional expression**	**Protein**	**Copies**
TP22	2	+	●	3	+	●	3
TP23	3	+	●	3	+	●	3
TP73	1	S	○	0	—	—	1
TP49	0	—	—	0	—	—	0
WT	Nontransgenic plants

“*S” indicates gene silencing; ●indicates protein expression; ○ indicates no protein expression*.

**Table 2:** Hybrid combination number, GUS activity, and multiplex PCR results.

**Hybrid combination**	**GUS expression**	**Multiplex PCR**			**RT- PCR**		
		***Gus***	***NptII***	***Bgt***	***Gus***	***NptII***	***Bgt***
TP22	●	+	+	+	+	+	+
TP73×TP22 (287)	◐	+	+	+	+	+	+
TP23×TP49 (116)	●	+	+	+	+	+	+
TP23	●	+	+	+	+	+	+
WT×TP23 (212)	◐	+	+	+	+	+	+
TP73×WT (9)	○	+	+	–	S	S	S
WT×TP22 (184)	●	+	+	+	+	+	+
TP22×TP22 (284)	◐	+	+	+	+	+	+
TP23×TP49 (196)	○	+	+	+	s	+	+
TP23×WT (29)	◐	+	+	+	+	+	+
TP73	○	+	+	–	s	s	–

●*Indicates GUS enzyme activity; ○indicates no GUS enzyme activity; ○ indicates partial GUS enzyme activity; “S” indicates gene silencing*.

### Detection of GUS Activity and Pollen Viability in Transgenic Birch Pollen

We collected the individual pollen of hybrid progeny plants of transgenic birch for GUS enzyme activity detection (Chong et al., 1998). GUS activity was detected using a histochemical method. The appropriate amount of pollen was added to a centrifuge tube, immersed in the GUS test solution (0.01 mg·mL^−1^), and incubated at 37°C for 1 h. After slight centrifugation, the test solution was removed and decolorized 2–3 times by adding 70% ethanol. The supernatant was removed by centrifugation. The pollen was suspended in an appropriate amount of deionized water. A small amount of pollen was dropped on the glass slide, covered with a coverslip, and observed under a microscope. The pollen was photographed and counted for separation of the *Gus* gene. Pollen viability was measured by the FDA method (Heslop-Harrison and Heslop-Harrison, 1970). The collected pollen was suspended in a staining solution containing 10% (w/v) Suc and 0.1 mg·mL^−1^ FDA and visualized under a fluorescence microscope (Zeiss Axio Scope A1, Germany).

### Multiplex PCR

Genomic DNA was isolated from fresh leaves using the CTAB method. Multiplex PCR was performed in a reaction volume of 30 μL, containing 50 ng DNA, 0.5 μM of each primer (Supplementary Table 1), 200 μM dNTPs, and 1 U Taq DNA polymerase. PCR was performed using the following program: 94°C for 3 min; 35 cycles of 94°C for 30 s, 58°C for 40 s, and 72°C for 1 min; and 72°C for 10 min. The amplification products were subjected to electrophoresis on 0.8% (w/v) agarose gel.

### ELISA of BGT Proteins

The Bradford protein concentration determination kit (Beyotime) was used to measure total protein content of the extracted birch leaves. BSA was used as a standard to obtain the standard curve of protein concentration. The curve was quantified as *y* = 0.0018*x* – 0.02, *R*^2^ = 0.9902. BGT protein solutions of various concentrations (37.5, 75, 150, 300, 450, 600, and 750 ng·mL^−1^) and a negative control were used to obtain the BGT-ELISA standard curve. A standard curve for the correspondence between BGT protein content and OD_450_ value was prepared. This curve was quantified as *y* = 0.0015*x* – 0.0694, *R*^2^ = 0.9879.

For BGT-ELISA, the protein samples were diluted 10 times with PBS for measurement. All measurements were repeated three times. The measurement results were fitted to a regression equation to calculate the corresponding BGT content. BGT protein content relative to the total soluble protein content was calculated based on BGT and total soluble protein contents of the samples.

### Insect Bioassay

Eggs of *Lymantria disar* were sterilized using 2% formalin solution for 3–5 min, rinsed with sterilized water, air dried, and placed in Petri dishes (Zeng et al., 2009). Newly hatched larvae were reared on artificial diets. Thirty newly molted (>24 h) second instar larvae were placed onto nontransgenic (control), transgenic, and hybrid birch leaves in a greenhouse under controlled conditions (16/8 h light/dark, 25 ± 2°C, 70–75% relative humidity). The larvae were weighed every 5 days. The insect bioassays were repeated three times.

### Methylation Analysis

Genomic DNA was subjected to bisulfite conversion using the EZ DNA Methylation-Gold™ Kit (Zymo Research, CA, USA). The hybrid combination number, GUS activity assay, and multiplex PCR results are shown in Table 2. Primers for bisulfite sequencing PCR (BSP) were designed using METhprimer. Primer information is presented in Supplementary Table 2. Kismeth (http://katahdin.mssm.edu/kismeth) was used to compare positive clone sequencing results with the reference genome (Gruntman et al., 2008). The CpG site methylation ratio in each amplified fragment was calculated. The methylation site model was drawn using MSR (http://www.msrcall.com/MSRcalcalate.aspx).

### Total RNA Extraction and qRT-PCR Analysis

The improved CTAB method was used to extract total RNA (Zeng et al., 2010a). RNA concentration was measured with NanoDrop, and first-strand cDNA was synthesized using a reverse transcription kit (Takara, Dalian, China). Fluorescent qPCR was performed on the Applied Biosystems 7500 real-time PCR system. The reaction conditions are 95°C for 30 s, 95°C for 5 s, 60°C for 34 s, 95°C for 15 s, 60°C for 1 min, and 95°C for 15 s, 40 cycles. All experiments were repeated three times. Quantitative data was obtained using the 2^−ΔΔCT^ method and processed with IBM SPSS 19.

## Results

### Exogenous Gene Integration and Expression in Transgenic Birch Pollen

**Figure 1:**
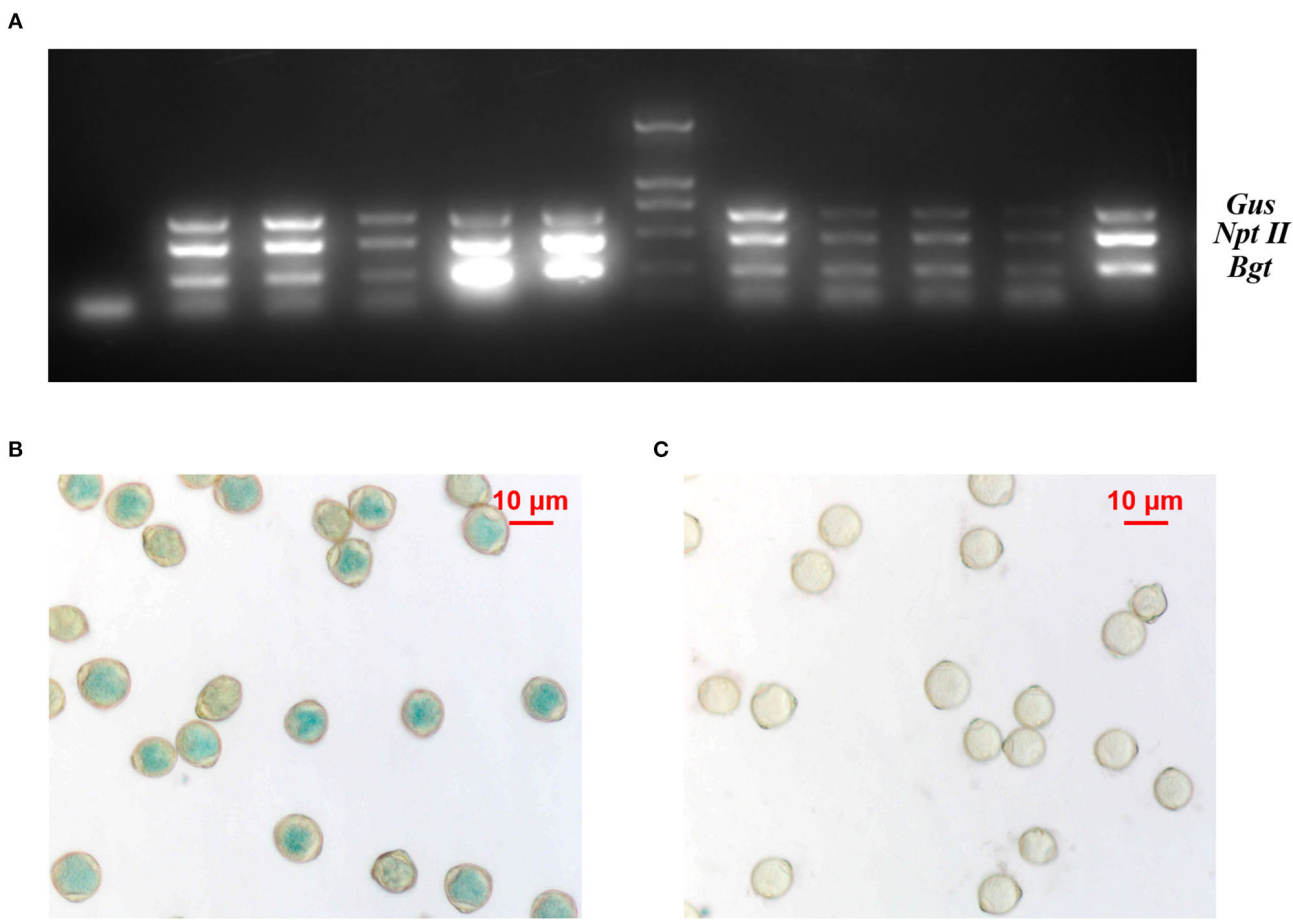
Analysis of transgenic birch pollen. **(A)** Multiplex PCR on total genomic DNA from transgenic birch pollen; 1: negative control; 2–6, 8–11 lanes, multiplex PCR on different clones of transgenic birch pollen DNA; M: DL2000 marker; 12: positive control. **(B)** Expression of *Gus* in TP23 transgenic white birch pollen. **(C)** Expression of *Gus* in nontransgenic birch pollen.

The pollen grains of different transgenic lines were viable and capable of pollination (Supplementary Figure 1). Multiplex PCR-based detection of different transgenic birch clones showed clearly amplified products of the three genes, indicating that the exogenous genes could be transferred in the pollen (Figure 1A). Although the *Gus* gene was normally expressed in transgenic birch pollen, not all pollen grains showed GUS enzyme activity, indicating that the exogenous genes were segregated in transgenic birch pollen during meiosis (Figures 1B,C).

### Inheritance and Expression of Exogenous Genes in Hybrid Progeny

**Figure 2:**
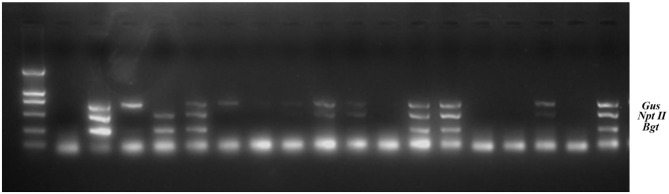
Multiplex PCR on total genomic DNA from transgenic birch progeny. M: DL2000 marker, 2: Negative control (wild type); 3: Positive control (DNA of recombinant plasmid). The remaining lanes indicate TP23 hybrid progeny samples.

Genomic DNA was extracted from the transgenic birch F1 progeny. Multiplex PCR and RT-PCR were performed to assess the inheritance and expression of the three exogenous genes (*Gus, NptII*, and *Bgt*) (Figure 2, Table 2). The results of histochemical staining and multiplex PCR of F1 progeny indicate that the exogenous gene could be stably inherited by the next generation through female or male gametes during transgenic birch hybridization. Transcriptional expression of exogenous genes in the hybrid progeny of TP22 and TP23 was normal. The expression of exogenous genes was silenced in all hybrid progeny of TP73 (Table 2). The exogenous genes were successfully integrated in the hybrid progeny of TP73×WT (9), but the corresponding RT-PCR amplification bands were not obtained, indicating that the exogenous genes were silenced at the transcriptional level (Table 2). The F1 seedlings were subjected to histochemical detection of GUS activity. The *Gus* gene showed enzymatic activity in the hybrid progeny, and there was no post-transcriptional silencing of exogenous genes.

### Transcription Levels of the Insect-Resistance Gene *Bgt*

**Figure 3:**
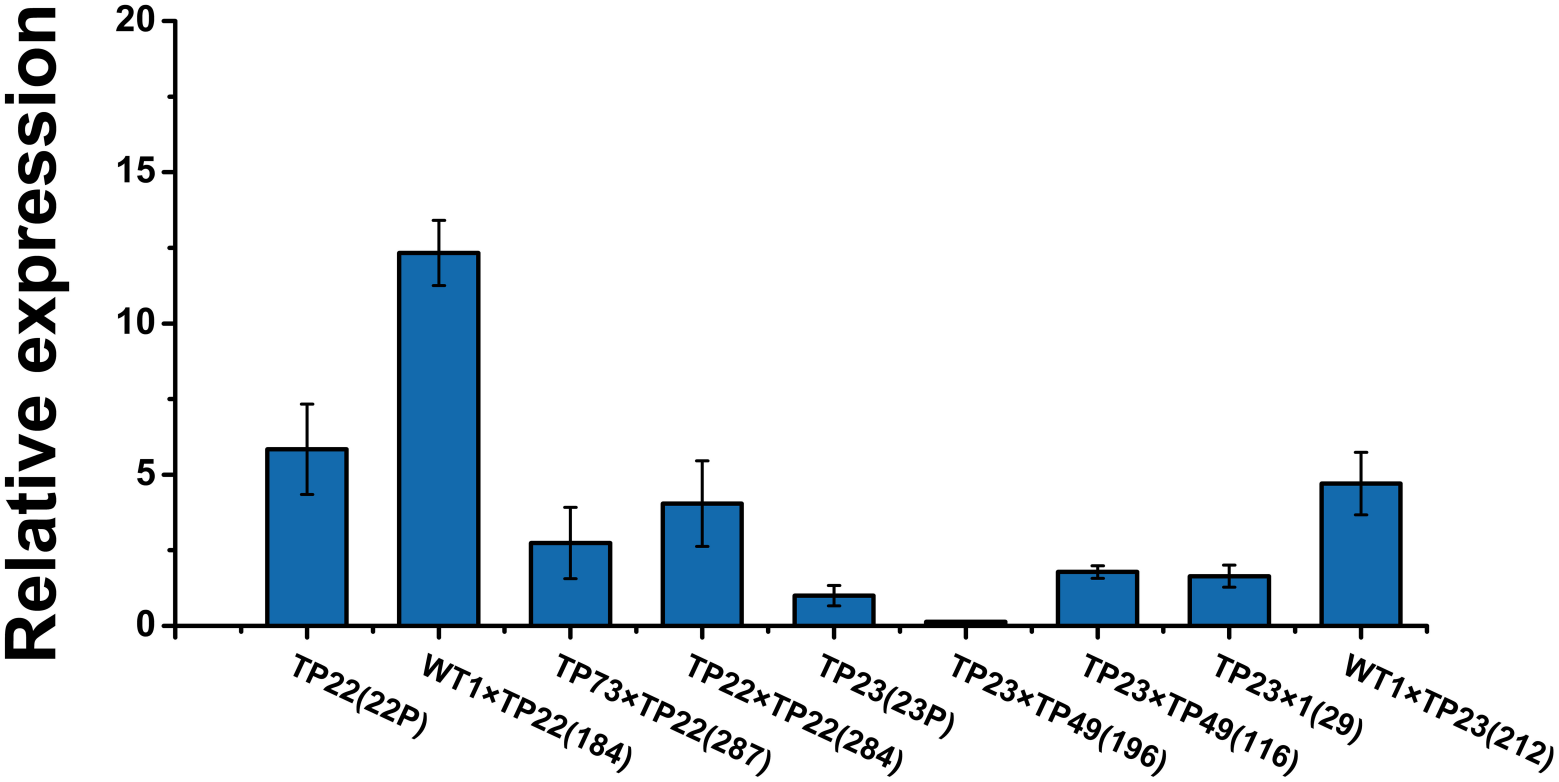
Relative expression of Bgt in F1 progeny. qRT-PCR analysis of *Bgt* expression in different transgenic birch samples. Error bars indicate standard deviation. TP22 (22P), WT1×TP22 (184), TP73×TP22 (287), TP22×TP22 (284), TP23 (23P), TP23×TP49 (196), TP23×TP49 (116), TP23×WT (29), WT1×TP23 (212). Data are presented as the mean ± SE of three biological replicates.

Transcription levels of the exogenous gene *Bgt* were examined in the transgenic parents and part of their F1 progeny. The average relative expression of *Bgt* in TP23 and its progeny was lower than that in TP22 and its progeny (Figure 3). Among TP22 and its F1 hybrid progeny, line 184 (WT1×TP22) showed transgressive expression, and the relative expression of *Bgt* in this line was ~2.11 times higher than that in its parents. The relative expression of *Bgt* in lines 287 (TP73×TP22) and 284 (TP22×TP22) was slightly lower than that in their respective parents (46.92 and 69.29%, respectively). The relative expression of *Bgt* in line 212 (WT×TP23) was 4.70 times higher than that in its parent TP23 and 2.86 times higher than that in line 29 (TP23×WT). The relative expression of *Bgt* in line 196 (TP23×TP49) was only 13.5% of the expression level in its parents. Therefore, the expression levels of *Bgt* varied across hybrid progeny with some hybrid lines showing higher expression than their parents.

### Protein Expression in and Insect Resistance of Transgenic Birch Hybrid Progenies

**Figure 4:**
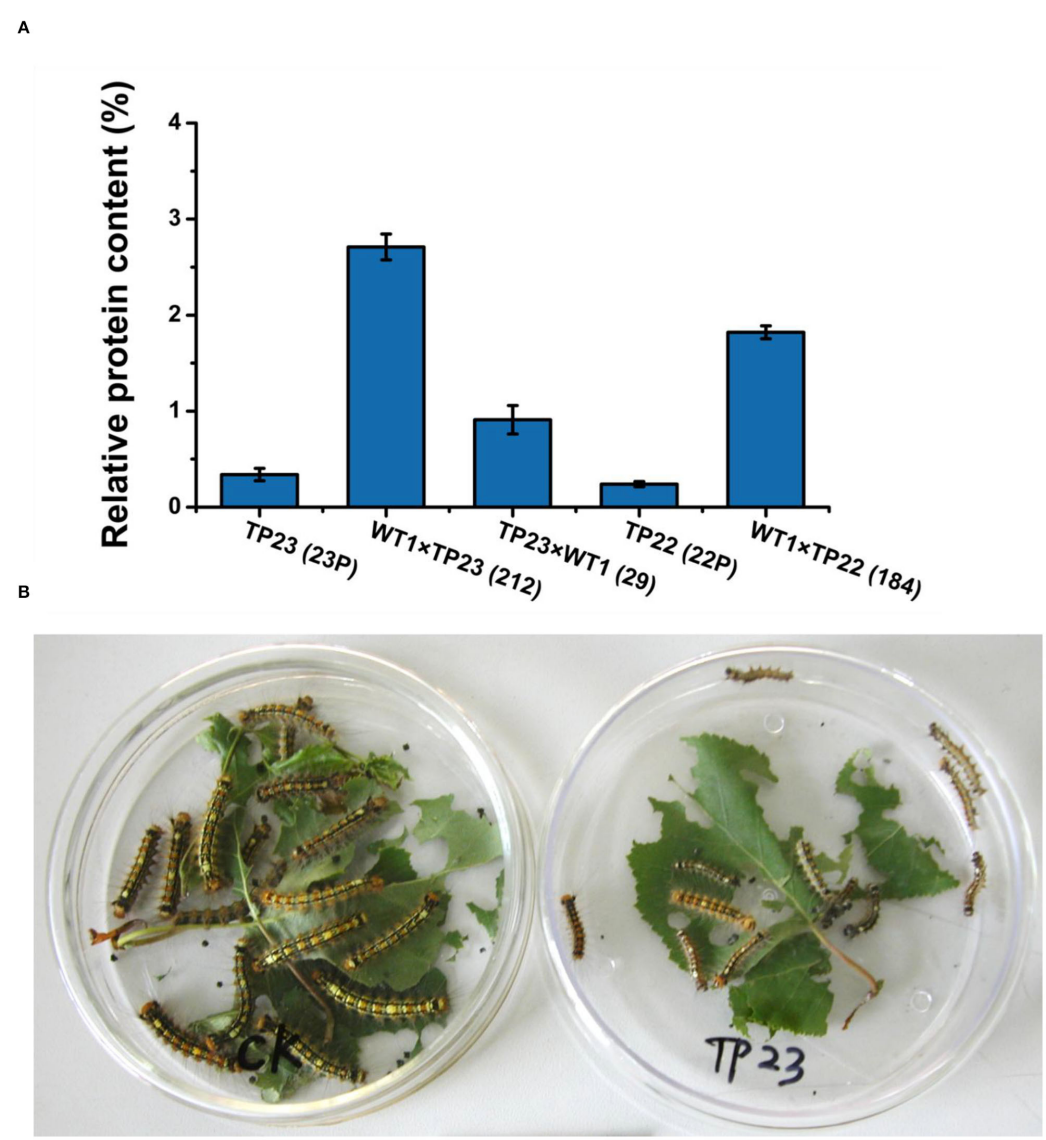
Protein level of the insect-resistance gene *Bgt*. **(A)** Comparison of BGT protein levels between the parents and progeny of transgenic birch. Error bars indicate standard deviation. TP23 (23P), WT1×TP23 (212), TP23×WT (29), TP22 (22P), WT1×TP22 (184). **(B)** Comparison of insect feeding results between the Control and transgenic birch TP23. Control larvae are indicated on the left side, and the larvae fed with TP23 are indicated on the right side. Data are presented as the mean ± SE of three biological replicates.

The expression levels of BGT proteins in the two hybrid lines, namely 212 and 284, were ~10 times higher than those in their parents and much higher than those in other hybrid lines (Figure 4A). These results show that the exogenous gene *Bgt* could normally express the protein in the hybrid progenies of transgenic birch.

The transgenic birch parents and their hybrid progeny were used to feed *Lymantria disar* larvae with nontransgenic birch plants as the control (Table 3, Figure 4B). On the 20th day of the experiment, the mortality rate of the larvae fed with hybrid lines 184, 212, and 29 was 100%, whereas the mortality rates of the larvae fed with progenies of TP22, TP23, and WT were 63, 62.5, and 22.5%, respectively. These results indicate that the transgenic birch progenies were highly insect resistant. The average weight of the surviving larvae was 20–50% that of their parent clones and 1–5% that of the control larvae. The results of insect bioassays were consistent with those of the BGT protein contents, which further confirmed that the exogenous genes could be expressed in F1 progeny of transgenic birch, and the expression levels of these genes in some hybrid progenies were significantly higher than those in their transgenic parents.

**Table 3:** Comparison of insect feeding results between parent and progeny of transgenic birch.

**Parent and progeny**		**WT**	**TP23**	**WT×TP23 (212)**	**TP23×WT (29)**	**TP22**	**WT×TP22 (184)**
13 d	Mortality rate (%)	15.0 ± 1.2	55.0 ± 1.43	75.0 ± 2.15	71 ± 1.68	47.5 ± 2.53	65.0 ± 4.34
	Corrected (%)	–	47.06 ± 0.86	70.59 ± 2.14		36.53 ± 1.76	58.82 ± 3.23
	Average weight (mg)	96.98 ± 2.31	31.24 ± 1.32	5.60 ± 0.26	6.20 ± 0.11	31.04 ± 1.23	6.37 ± 0.48
20 d	Mortality rate (%)	22.50 ± 1.42	62.50 ± 0.32	100.00	100.00	63.00 ± 3.12	100
	Corrected (%)	–	51.61 ± 0.58	100.00	100.00	54.84 ± 1.57	100
	Average weight (mg)	221.10 ± 5.60	165.71 ± 4.35	–	–	156.80 ± 7.54	–
30 d	Mortality rate (%)	27.5 ± 1.45	97.5 ± 2.11	–	–	85.0 ± 3.23	–
	Corrected (%)	–	96.55 ± 1.48	–	–	79.31 ± 2.15	–
	Average weight (mg)	566.47 ± 6.89	203.23 ± 5.47	–	–	203.85 ± 6.75	–

### DNA Methylation of *Bgt* and *Gus*

**Figure 5:**
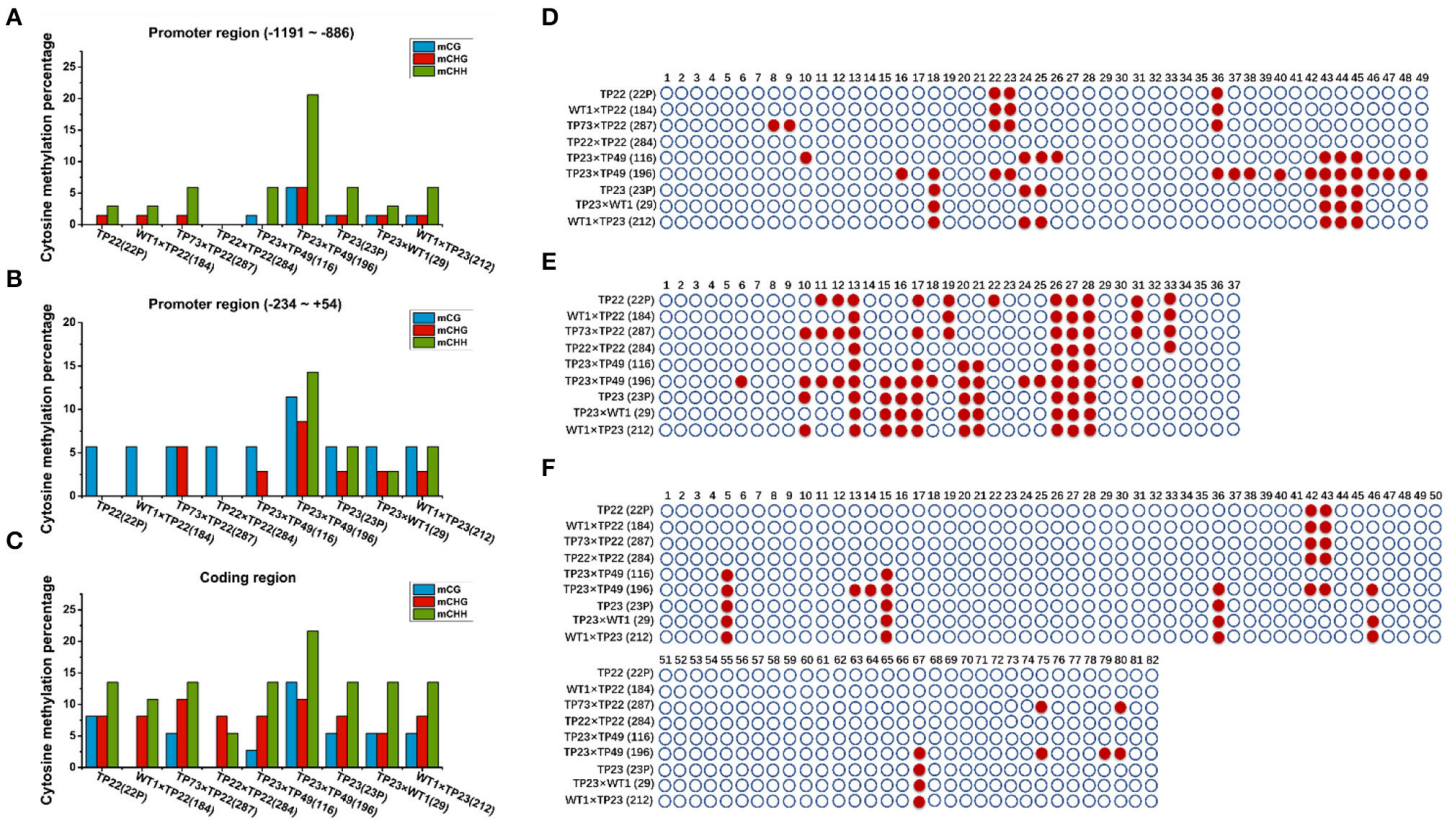
Cytosine methylation of *bgt*. **(A–C)** Cytosine methylation percentage of *Bgt* in parents and hybrid progeny. **(D)** *Bgt* promoter −1,191 to −886 region. **(E)** *Bgt* coding region. **(F)** *Bgt* promoter −234 to +54 region. CG and CNG are cytosines with a symmetric site. CHH is an asymmetric site for cytosine. TP22, WT1×TP22 (184), TP73×TP22 (287), TP22×TP22 (284), TP23×TP49 (116), TP23×TP49 (196), TP23 (23P), TP23×WT (29), WT1×TP23 (212). **(D–F)** Methylation status analysis. Each row represents different parents and progeny. TP22, WT1×TP22 (184), TP73×TP22 (287), TP22×TP22 (284), TP23×TP49 (116), TP23×TP49(196), TP23, TP23×WT1(29), WT1×TP23(212). ○ Represents nonmethylated cytosine in CG/CHH/CHG, and ● represents methylated cytosine. **(D)** *Bgt* promoter −1,191 to −886 region. **(E)** *Bgt* promoter −234 to +54 region. **(F)** *Bgt* coding region.

Cytosine methylation rates in the promoter regions in the *Gus* gene–silenced line 196 were significantly higher than those in the nonsilenced lines (Figure 5). The methylation rates in the coding regions were significantly higher than those in the promoter regions of *Bgt* in all plants (Figures 5A–C). However, the expression of the exogenous genes was not affected, indicating that the effect on gene silencing was realized through methylation in the promoter region rather than in the coding region. The methylation rate in the coding region of *Bgt* in line 184 was also lower than that in its parent TP22 (Figure 5C), indicating the occurrence of demethylation in this region. However, the methylation rates did not significantly differ between other hybrid lines and their parents.

The 20th base in TP23 and its progeny, but not in TP22 and its F1 progeny (Figures 5D–F), was a significant methylation site, indicating that the methylation sites in the promoter and coding regions of the *Bgt* gene could be stably inherited by the next generation through sexual reproduction. The methylation rates in the promoter and coding regions of the *Bgt* gene in line 284 were lower than those in its parents, indicating that the methylation pattern was reconstructed in this hybrid progeny. Compared with TP23, the hybrid line 29 (TP23×WT) showed demethylation at two sites in the promoter (−1,191 to −886 and −234 to +54) region of the *Bgt* gene. The methylation rate in line 196 (TP23×TP49) was greater than that in its parent TP23 (promoter regions −234 to +54 and −1,911 to −886 and the coding region).

**Figure 6:**
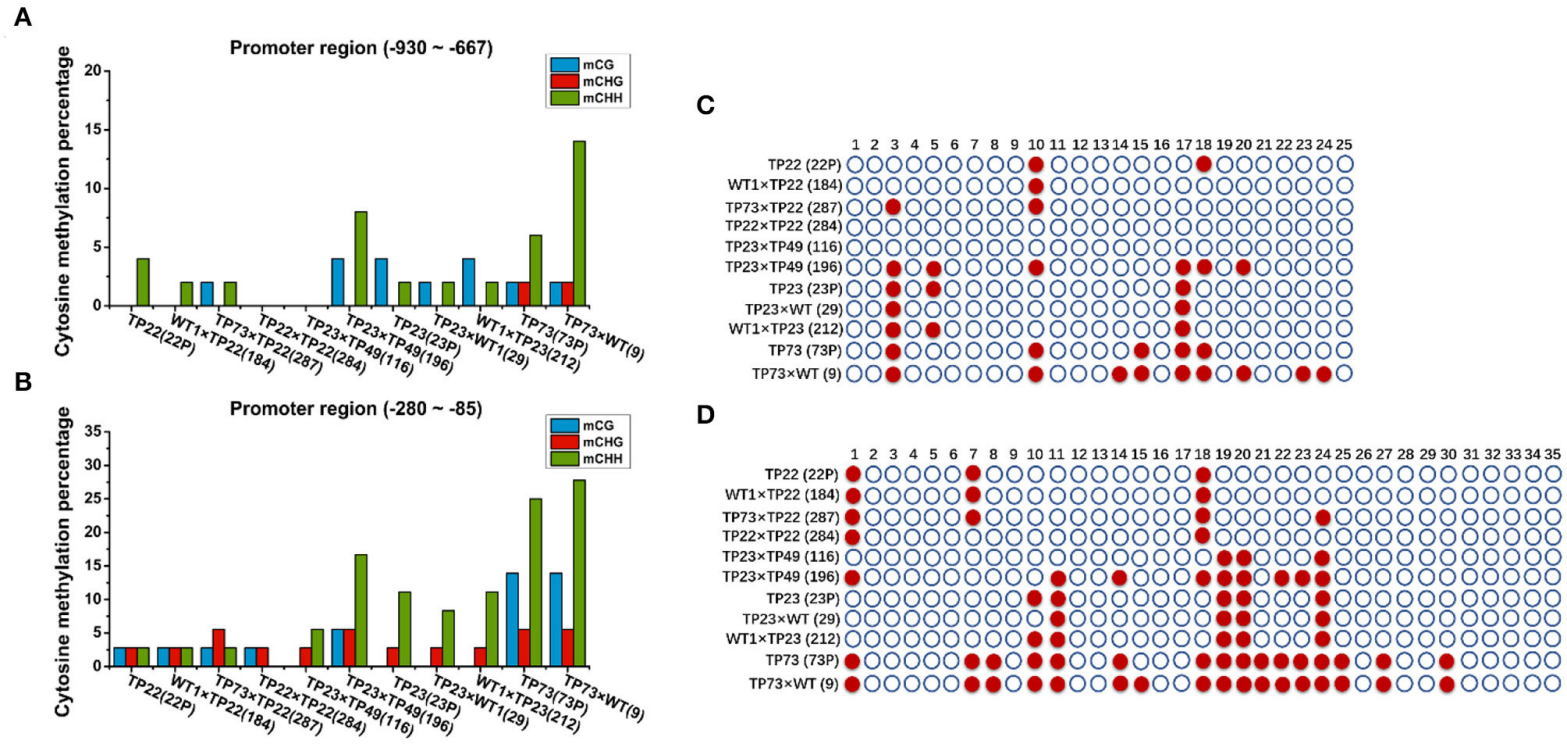
Cytosine methylation of *Gus*. **(A,B)** Cytosine methylation percentage of *Bgt* in parents and hybrid progeny. **(A)** *Gus* promoter −930 to −667 region. **(B)** *Gus* promoter −280 to −85 region. **(C,D)** Methylation status analysis. Each row represents different parents and progeny. TP22 (22P), WT1×TP22 (184), TP73×TP22 (287), TP22×TP22 (284), TP23×TP49 (116), TP23×TP49 (196), TP23, TP23×WT (29), WT1×TP23 (212), TP73 (73P), TP73×WT (9); ○ represents nonmethylated cytosine in CG/CHH/CHG, and ● represents methylated cytosine. **(C)** *Gus* promoter −930 to −667 region. **(D)** *Gus* promoter −280 to −85 region.

To further demonstrate the effects of methylation in the promoter regions on gene silencing, we examined the methylation rate in the *Gus* promoter (Figures 6A,B). The distal cytosine methylation rate in the *Gus* promoter was 4% in TP22, 2% in its hybrid progeny 184 (WT×TP22), and 0% in its hybrid progeny 284 (TP22×TP22). The proximal cytosine methylation rate in the *Gus* promoter was 8.33% in TP22, 8.33% in its hybrid progeny 184, and 5.56% in its hybrid progeny 284. The distal cytosine methylation rate in the *Gus* promoter was 6% in TP23 and 12% in its hybrid progeny 196. The cytosine methylation rate was below 6% in the remaining hybrid progenies. The cytosine methylation rates in the *Gus* promoter of the gene-silenced plant TP73 and the hybrid progeny 9 and 196 were significantly higher than those in nonsilenced plants, suggesting that the level of promoter cytosine methylation is closely related to *Gus* gene silencing. TP23 harbors the same methylation site as its hybrid progeny 29, and TP22 also harbors the same methylation site as its progeny (Figures 6C,D). These results indicate that the methylation site is stable and inherited. The exogenous genes were silenced in TP73. TP73 also harbored significantly more methylation sites than normal transgenic birch parents, and its progeny inherited these methylation sites. Meanwhile, the hybrid lines 184, 116, and 284 harbored fewer methylation sites in the *Gus* gene promoter region −280 to −85 than their parents.

### Expression of *BpCMT, BpMET*, and *BpDRM* in Transgenic Birch

**Figure 7:**
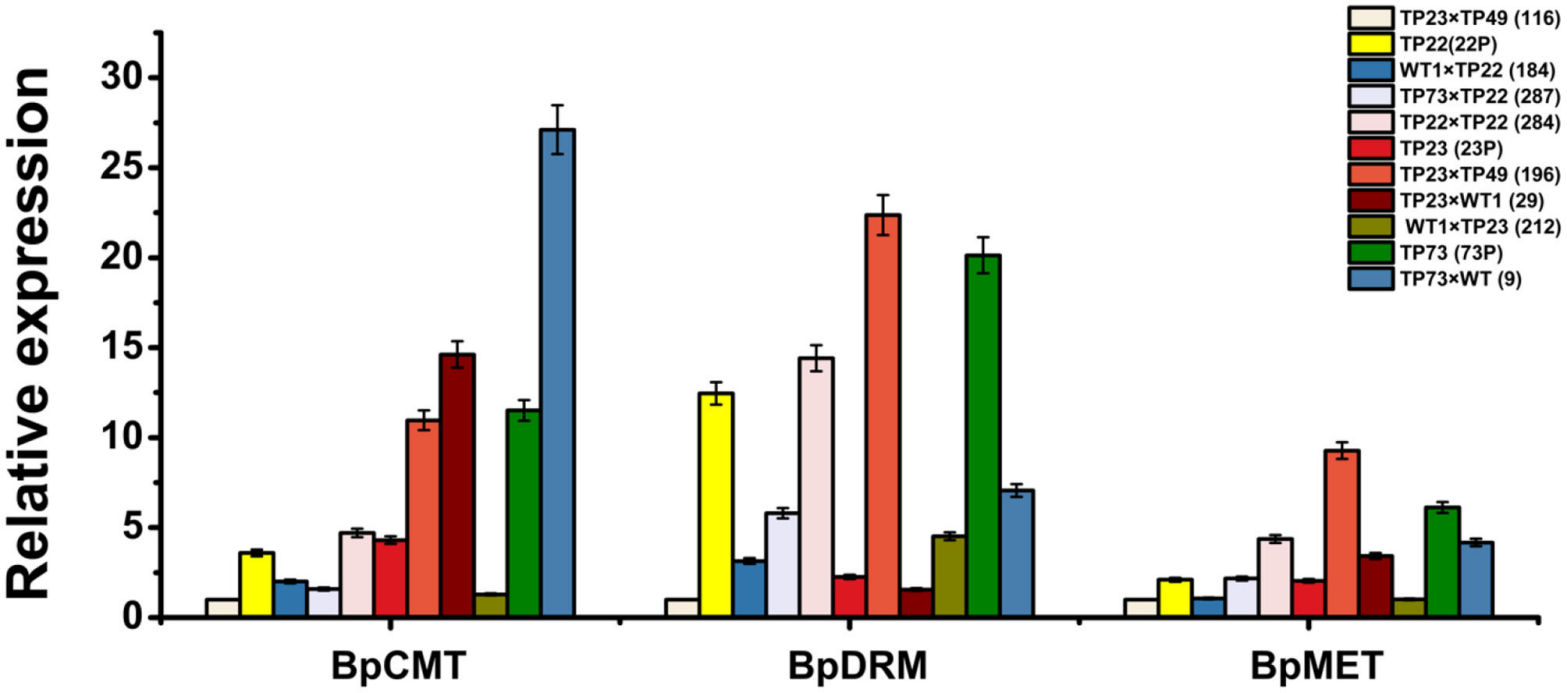
Relative expression of the methyltransferase gene in parents and progeny of transgenic birch. qRT-PCR analysis of *BpCMT, BpDRM*, and *BpMET* expression in different transgenic birch. Data are presented as the mean ± SE of three biological replicates. Error bars indicate standard deviation.

To further investigate the association between exogenous gene silencing and methylation, the expression of the methyltransferase genes *BpCMT, BpMET*, and *BpDRM* was examined. Gene expression was the lowest in line 116; thus, it was used as a control to analyze the relative expression levels of methyltransferase genes (Figure 7). There was an obvious difference in the relative expression levels of the three methyltransferase genes between TP22 and its hybrid progeny. The relative expression levels of *BpCMT, BpDRM*, and *BpMET* in TP22 were, respectively, 3.59, 12.46, and 2.10 times higher than those in its hybrid progenies (184 and 287). The relative expression levels of the three methyltransferase genes in the self-crossed line 284 were slightly higher than those in its parent (TP22). The relative expression level of the *Bgt* gene was higher in plants with lower relative expression levels of methyltransferase genes (Figures 3, 7).

Among TP23 and its progeny, the relative expression levels of the three methyltransferase genes were the lowest in line 116. The relative expression of *BpCMT* in line 29 was 3.39 times the expression in its parent. The relative expression of *BpMET* in the progeny was slightly higher than that in the parent, whereas the relative expression of *BpDRM* was slightly lower than that in the parent. The relative expression levels of *BpCMT, BpDRM*, and *BpMET* in the *Gus* gene–silenced line 196 were, respectively, 2.54, 9.92, and 4.54 times higher than those in its parents. These trends are consistent with hypermethylation in the promoter and coding regions of the exogenous genes. The expression levels of *BpCMT, BpDRM*, and *BpMET* in TP73 were, respectively, 2.67, 8.93, and 3 times higher than those in TP23 and, respectively, 3.21, 1.62, and 2.91 times higher than those in TP22. The relative expression levels of *BpDRM* and *BpMET* genes in line 9 (TP73×WT) were significantly lower than those in TP73, whereas the expression level of *BpCMT* in line 9 was 2.11 times higher than that in TP73.

Correlation analysis showed a negative correlation between the expression of the *Bgt* and methyltransferase genes (Table 4). There was a positive correlation among the expression levels of the methyltransferase genes. In summary, high expression levels of methyltransferase genes led to hypermethylation of exogenous genes and suppressed *Bgt* expression in transgenic birch plants.

**Table 4:** Correlation between the expression of *Bgt* and methyltransferase genes.

	***Bgt***	***BpCMT***	***BpDRM***	***BpMET***
*Bgt*	1.0000			
*BpCMT*	−0.4332	1.0000		
*BpDRM*	−0.1856	0.2943	1.0000	
*BpMET*	−0.4646	0.6683	0.8393	1.0000

## Discussion

### Expression and Stability of Exogenous Genes in Hybrid Progeny

Exogenous genes should be normally transmitted to the offspring. During meiosis, exogenous genes can be normally assigned to the male or female gametes. Many studies show that the presence of transgenes may affect the development and maturation of gametes, leading to the inability of transgenes to be efficiently transmitted to the progeny through gametes, resulting in abnormal segregation of the transgenes. Sangtong et al. (2002) find that the wheat *Glu-1DX5* gene could not be efficiently transmitted to transgenic maize through pollen. Aragão et al. (1996) find that the introduction of an exogenous gene may induce insertional mutations in genes that are necessary for fertilization and development of egg cells, resulting in abnormal segregation of the *Gus, neo, AC123*, and *BC1* genes in transgenic beans. Limanton-Grevet et al. (2000) also find that exogenous genes could only be transmitted through male gametes, perhaps due to insertional mutations affecting the viability of female gametes. Studies have shown that the genetic characteristics of exogenous genes are related to transformation methods (Gerszberg, 2018). Currently, most transgenic plants are mainly obtained through *Agrobacterium*-mediated transformation and the gene gun method. *Agrobacterium*-mediated gene transformation has the advantages of single integration and genetic stability of exogenous genes, whereas gene gun transformation allows for the integration of multiple exogenous gene copies, but their expression is unstable (Lee and Zhang, 2013). The pollen of both transgenic and nontransgenic birch plants was viable. Multiplex PCR also showed that the exogenous genes were inherited by hybrid offspring, which initially revealed the possibility of exogenous gene transmission through pollen. Detection of GUS activity further indicates that the reporter gene could be normally expressed in pollen. Quantitative analysis of the *Bgt* gene in parents and hybrid progeny showed that the expression level of the *Bgt* gene in progeny was variable although most of the hybrid lines showed higher expression levels than their parents. In previous studies, the *Bgt* gene was found to have been silenced at the transcriptional level in both single- and multi-copy plants, indicating that there is no obvious association between the silencing of exogenous genes at the transcriptional level and their copy numbers (Zeng et al., 2009).

### Cytosine Methylation Patterns and Stability of Exogenous Genes

During the growth and development of plants, DNA methylation plays important roles in gene expression, defense, and cell development and differentiation. The methylation status of parental plants is usually stably inherited by the next generation (Cubas et al., 1999). Epigenetic information is based on changes in DNA methylation or chromatin status and is usually heritable during cellular reproduction, particularly in plants, in which the epigenetic status of genes affecting phenotypic traits is inherited over generations (Henderson and Jacobsen, 2007). By analyzing the specific methylation sites in the coding and promoter regions of *Gus* and *Bgt*, we find that the methylation sites in parents and progenies in the same family are highly similar, whereas there are differences in methylation sites across different families (Figure 5). However, the absence of methylation at the 42nd base in the *Bgt* promoter in line 116 was different from the methylation site in the parent, which may be caused by self-demethylation (Figure 5F). Our results are consistent with the results reported by Zhao et al. (2007) regarding methylation levels and patterns in maize hybrids. Fulneček et al. (2009) also studied tetraploid tobacco and find that the inherited CG and CHG methylation sites were highly similar to the parental sites, and the CHH methylation site was altered during reproduction. The methylation sites share high similarity between parents and offspring, indicating that the parents can pass the methylated genetic loci and genetic information to the offspring through sexual reproduction. Messeguer et al. (1991) demonstrate the stable Mendelian inheritance of methylated polymorphisms in the parents by the offspring. In plants, DNA methylation mainly occurs at the symmetric CG and CNG cytosines, and 5-mC appears symmetrically in double-stranded DNA fragments harboring CG and CNG, which can ensure the inheritance of this modification (Kakutani et al., 1996). These results indicate that DNA methylation patterns can be stably transmitted to the offspring by gametes through sexual reproduction.

Presumably, hypermethylation of the promoter and coding regions in the plant genome inhibits gene transcription and leads to gene silencing by preventing the binding of the transcription factor complex to DNA (Wendte et al., 2019). Genomic studies in *Arabidopsis* reveal that many endogenous genes are methylated in their promoter or coding regions, and gene methylation levels are strongly correlated with transcription levels (Vaughn et al., 2007; Cokus et al., 2008). DNA methylation occurs mainly in the CpG island-rich promoter region, which may hinder the binding of transcription factors to the promoter, thereby inhibiting gene transcription (Zhai et al., 2019). The methylation pattern of exogenous genes reveals that the methylation rate of the CG site of the *Gus* promoter (−280 to −85 region) is 0–14.28% and that of the CHG sites is 33.33–66.67%. The overall methylation rate at the symmetric cytosine sites (CHG and CG) is ~10–30%. The methylation rate at the CHH site in this region is 3.8–15.38%. In plants with the silenced *Gus* gene, the methylation rate at the CG site in the *Gus* promoter (−280 to −85 region) is 28.57–71.43% and that at the CHG site is 66.67%. The methylation rate at the nonsymmetric cytosine site CHH is 23.08–38.46%. In the *Bgt* gene, the rate of methylation at the symmetric cytosine sites is higher than that at the asymmetric cytosine sites. Our results indicate that the methylation patterns of exogenous genes can be inherited through sexual reproduction.

### Relationship Between DNA Methylation and Exogenous Gene Silencing in Plants

DNA methylation is associated with gene silencing (Law and Jacobsen, 2010; Lang et al., 2015; Yang et al., 2018). Plant gene silencing is divided into TGS and post-transcriptional gene silencing (PTGS) (Fagard and Vaucheret, 2000). When the homology between the interacting genes is confined to the coding regions, it often leads to PTGS. When this homology is confined to the promoter regions or includes promoter sequences, it leads to TGS (Bologna and Voinnet, 2014). Transgene integration stability and expression levels in long-term micropropagation clones of transgenic birch were examined in a previous study (Zeng et al., 2010a). The transcriptional expression levels of exogenous genes in regenerated plants decreased with an increasing number of subcultures, indicating the occurrence of TGS in regenerated transgenic lines. In micropropagated transgenic birch, the expression of exogenous GUS and BGT proteins could be reactivated with Azac treatment, suggesting that the decrease in expression level with the increase in the number of subcultures is associated with DNA methylation (Zeng et al., 2010a).

Methyltransferases play pivotal roles in methylation maintenance and remethylation. *MET1* is a conserved key DNA methylase responsible for maintaining CG methylation in plants. A mutation of *MET1* led to DNA hypomethylation in the CG context in both *Arabidopsis thaliana* and rice (Hu et al., 2014; Yang et al., 2019). The correlation coefficient between *BpMET* expression and the methylation level of the *Bgt* promoter region is the greatest (Supplementary Table 3). The expression level of *BpMET* is highly correlated with the methylation level of the *Gus* promoter (*p* < 0.01) (Supplementary Table 4). In *Arabidopsis thaliana*, Chromomethylase3 mainly maintains CHG methylation, and Chromomethylase2 maintains CHH methylation in the chromosome arms and pericentromeric regions (Wang et al., 2015). The methylation level of the proximal *Bgt* promoter region and *Bgt* expression are significantly correlated (*p* < 0.05). Overexpression of methyltransferase genes in transgenic birch results in hypermethylation of exogenous genes and their subsequent silencing (Jones et al., 1999; Moritoh et al., 2012; Lang et al., 2015).

## Conclusion

Exogenous genes of transgenic birch can be transmitted to progeny through sexual reproduction. The expression of an exogenous *Bgt* gene significantly differed between parents and their hybrid progenies such that the expression levels of *Bgt* in most F1 hybrid lines were higher than those in their parents. The hybrid progeny of transgenic birch exhibited excellent insect resistance. The methylation sites of the exogenous genes could be inherited by the progeny through sexual reproduction. Transgene silencing in the progeny was mostly caused by DNA methylation at the cytosine. DNA methylation in the promoter region, rather than in the coding region, led to exogenous gene silencing. The expression of the *Bgt* gene was negatively correlated with the expression of methyltransferase genes. Here, we elucidate the factors affecting the genetic stability of transgenes in woody plants and provide a theoretical basis for the selection and breeding of stable and excellent insect-resistant birch trees.

## Data Availability Statement

The raw data supporting the conclusions of this article will be made available by the authors, without undue reservation, to any qualified researcher.

## Author Contributions

FZ and YZ conceived and designed the experiment. Experiments were conducted with MM, BL, XC, YY, and RF. MM and XC analyzed these data. FZ, MM, and XC wrote this paper. All authors contributed to the article and approved the submitted version.

## Conflict of Interest

The authors declare that the research was conducted in the absence of any commercial or financial relationships that could be construed as a potential conflict of interest.

## Abbreviations

Bgt: Spider Insecticidal Peptide and Bt-toxin C Peptide Gene
GUS: β-glucuronidase
FDA: Fluorescein diacetate
TGS: Transcriptional gene silencing
MET: methyltransferase
CMT: chromomethylase
DRM: domain-rearranged methyltransferase
qRT-PCR: quantitative reverse transcription polymerase chain reaction.
