# Supplementary material for: DNA Methylation Silences Exogenous Gene Expression in Transgenic Birch Progeny
Source: Front Plant Sci. 2020 Dec 22;11:523748. doi: 10.3389/fpls.2020.523748 (PMC7783445; doi:10.3389/fpls.2020.523748)
Supplement: Supplementary file 1 [file Data_Sheet_1.docx]

Supplementary Material

## Supplementary Figures


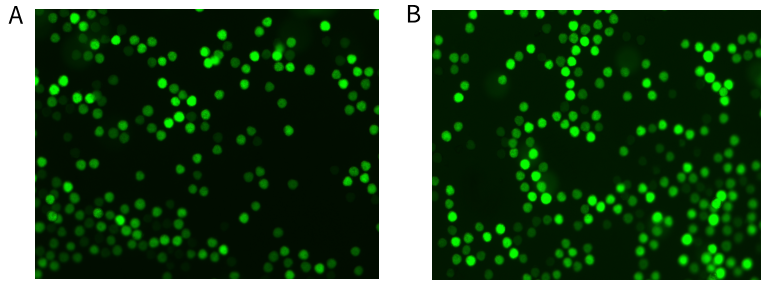


**Supplementary Figure 1.** Comparison ofon pollen viability in between transgenic (**A**) and non-transgenic (**B**) birch plants using fluorescein diacetate staining

## 2 Supplementary Tables

**Supplementary Table 1.** Related primers for multiplex PCR

| Name | Sequence | Length/bp | Tm/℃ | GC/% | Product length /bp |
| --- | --- | --- | --- | --- | --- |
| *BGT*-F | 5’AACGGTAGATTCGCTGGAT3’ | 19 | 54. 5 | 47. 4 | 247 |
| *BGT*-R | 5’CAGAAGTTCCAGAGCCAAG3’ | 19 | 52. 7 | 52. 6 |  |
| *NPT*II-F | 5’TCCGGCCGCTTGGGTGGAGA3’ | 20 | 59. 8 | 58. 3 | 449 |
| *NPT*II-R | 5’TGGCGCGAGCCCCTGATGCT3’ | 20 | 58. 7 | 58. 8 |  |
| *GUS*-F | 5’GCAACTGGACAAGGCACTA3’ | 19 | 53. 9 | 52. 6 | 668 |
| *GUS*-R | 5’AGCGTCGCAGAACATTACA3’ | 19 | 54. 7 | 47. 4 |  |

| Position | Name | Sequence (5’-3’) | base/bp | Tm/℃ | CG/% | Methylation site | | | Length/bp |
| --- | --- | --- | --- | --- | --- | --- | --- | --- | --- |
|  |  |  |  |  |  | CG | CNN | CNG |  |
| *Bgt* promoter distal region | B1-F | GATAGATAGTTGGGTAATGGAATT | 24 | 54. 77 | 50 | 10 | 51 | 7 | 306 |
|  | B1-R | CAAACTTACCAACATAATAAAACAC | 25 | 54. 23 | 56 |  |  |  |  |
| *Bgt* promoter proximal region | B2-F | GTGTAGGATTGGGAATAGTAATTTT | 25 | 56. 16 | 48 | 11 | 21 | 3 | 289 |
|  | B2-R | CCTCTATATAAAAAAATTCATTTCATTTAA | 30 | 57. 39 | 33. 33 |  |  |  |  |
| *Bgt* coding region | B3-F | TTTGTTTAGTTGTAATTATTTGTTGATT | 28 | 56. 17 | 50 | 12 | 19 | 6 | 162 |
|  | B3-R | AAAAAAACAATCTTACTTCCATAATTTCTT | 30 | 59. 65 | 33. 33 |  |  |  |  |

**Supplementary Table 2.** BSP amplification primer information.

Note: N stands for A, T, C; C in CG and CNG is a cytosine with a symmetric site; CNN is an asymmetric site for cytosine.

**Supplementary Table 3.** Correlation analysis between methylation level and methyltransferase

|  | *BpCMT* | *BpDRM* | *BpMET* | *bgt* |
| --- | --- | --- | --- | --- |
| Promoter distal region methylation level | 0. 4120 | 0. 5830 | 0. 7759 | -0. 4551 |
| Promoter proximal region methylation level | 0. 4796* | 0. 5583 | 0. 7962** | -0. 5517* |
| *Bgt* coding region methylation level | 0. 3574** | 0. 5345** | 0. 6334** | -0. 4586** |

Correlation analysis between methylation level and methyltransferase expression of *bgt* (* p < 0.05; ** p < 0. 01)

**Supplementary Table 4.** Correlation analysis between methylation level and methyltransferase

|  | *BpCMT* | *BpDRM* | *BpMET* |
| --- | --- | --- | --- |
| Promoter distal region methylation level | 0. 7230 | 0. 0828 | 0. 2516 |
| Promoter proximal region methylation level | 0. 7190 | 0. 2332 | 0. 3332** |

Correlation analysis between methylation level and methyltransferase expression of *gus* (* p < 0.05; ** p < 0. 01).
